# Supplementary material for: Spatial scale of receptive fields in the visual sector of the cat thalamic reticular nucleus
Source: Nat Commun. 2017 Oct 6;8:800. doi: 10.1038/s41467-017-00762-7 (PMC5630618; doi:10.1038/s41467-017-00762-7)
Supplement: Supplementary file 1 — Supplementary Information [file 41467_2017_762_MOESM1_ESM.pdf]

File Name: Supplementary Information
Description: Supplementary Figures, Supplementary Tables and Supple

File Name: Peer Review File

SUPPLEMENTARY MATERIALS

Supplementary Figure 1

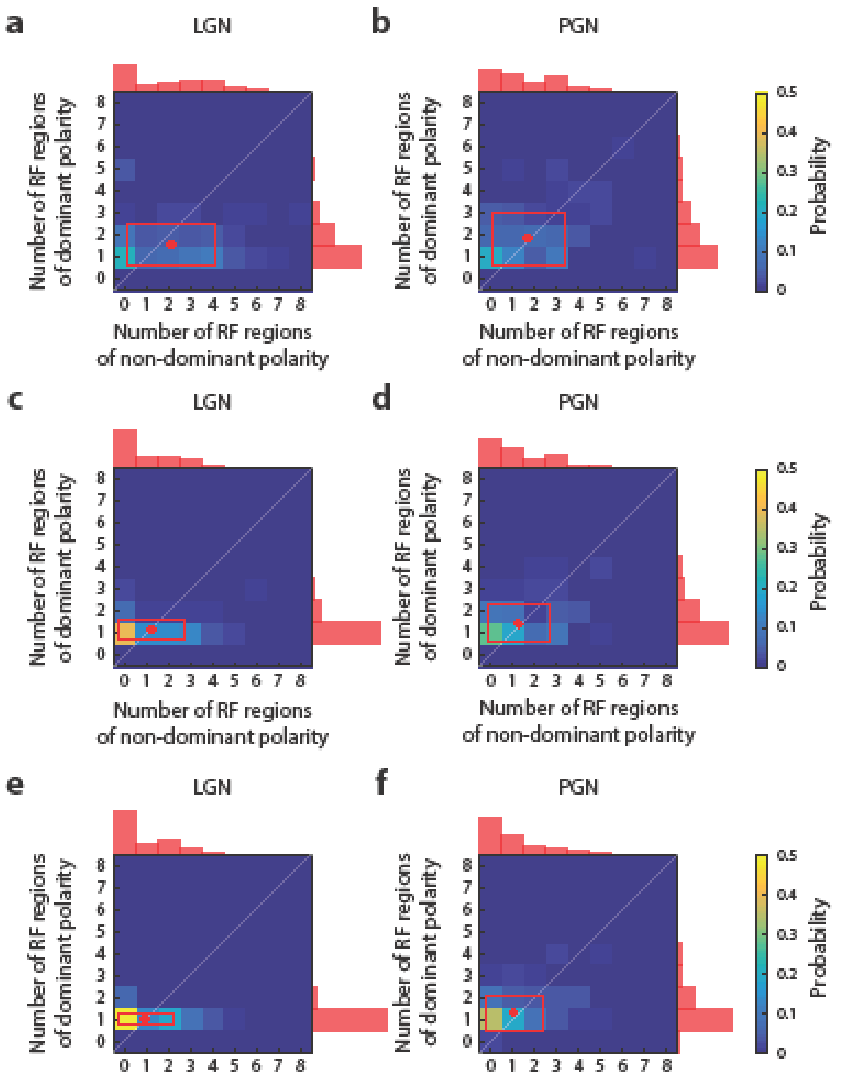

Supplementary Figure 1. *Spatially discrete regions in footprints for cells in the LGN and PGN.* The number of contiguous regions in footprints for LGN (left column) and PGN (right column); counts of regions of the dominant and non-dominant sign plotted against each other. The three rows of plots were made using thresholds of different stringencies. For panels **a** and **b**, $q = 0.05$; for panels **c** and **d**, $q = 0.01$; for panels **e** and **f**, $q = 0.001$. For each panel, the number of contiguous regions in the footprint for the dominant polarity is plotted against that for the non-dominant polarity, with the color code representing the frequency at which the pertinent numbers of RF regions were observed. Marginal histograms at the top and side of each plot show distributions for footprints of the dominant versus non-

3 Supplementary Figure 2

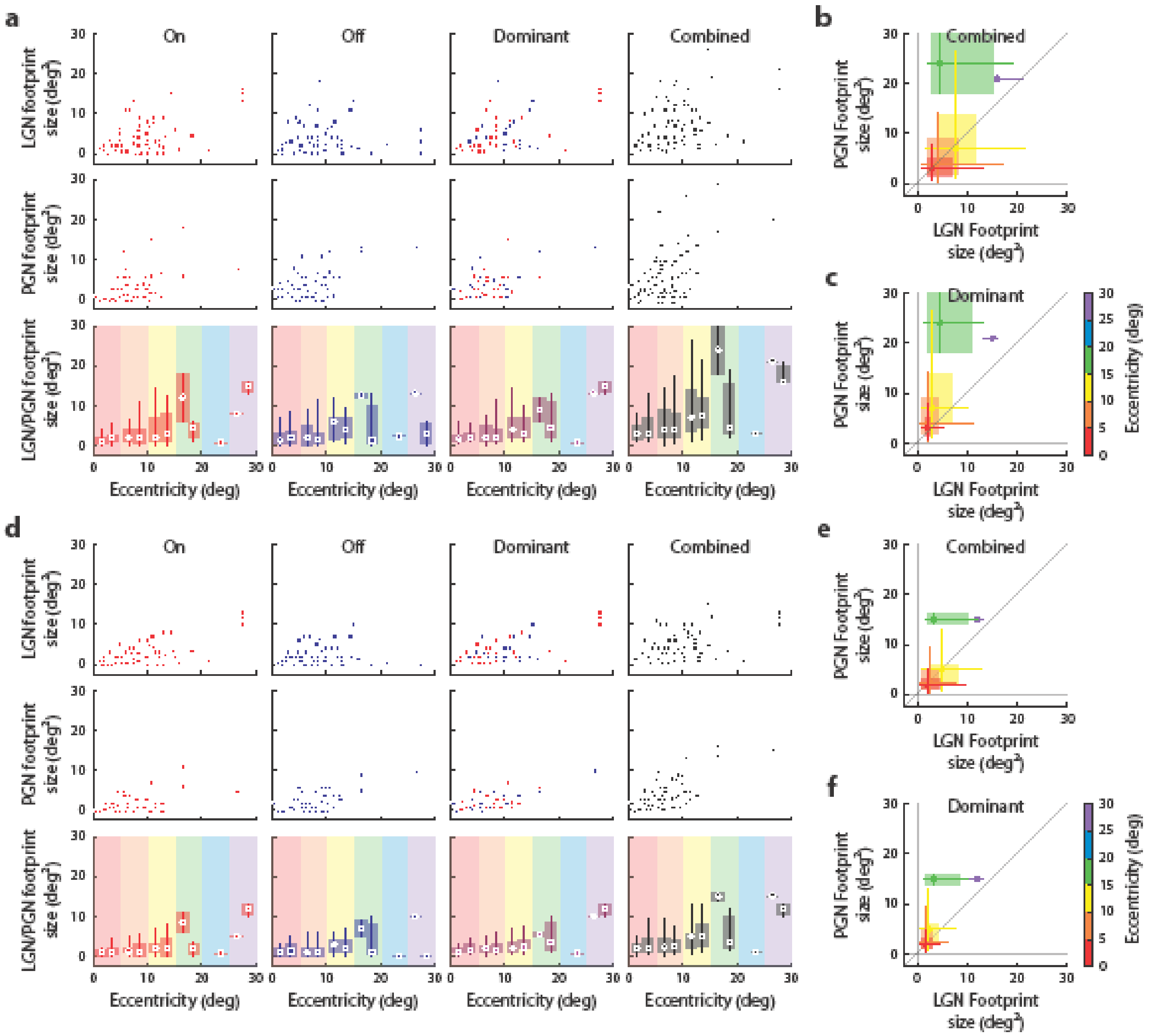

Supplementary Figure 2. *Footprints of cells in the LGN and PGN calculated using different threshold levels*. Conventions are as for Fig. 4 in the main text. Panels **a-c** in this figure were made from footprints generated with threshold, q = 0.05; panels **d-f** were generated with threshold, q = 0.001.

Estimate of cell number in the PGN

To address the questions of whether or not there might be enough cells in the PGN to cover visual space at a scale comparable to the LGN, we used data from the literature and our own measurements of the borders of the PGN and LGN from Nissl stained material to estimate the number of cells in the PGN. We then compared that estimate with accounts of the number of Y ganglion cells in retina.

Supplementary table 1 summarizes counts of different types of ganglion cells in the cat's retina. Supplementary table 2 summarizes the nuclear volume, cell densities and cell counts in the cat's visual thalamus, with measurements from previous reports and from our own laboratory. In conclusion, the calculations based on our data as well as on previous anatomical studies suggest that the total number of neurons in the PGN ($\sim 1.55 \times 10^4$) is 2-3 times greater than that of alpha (Y-type) retinal ganglion cells (6.7×10^3) in the cat.

Supplementary Table 1

Counts of retinal ganglion cells in the cat

Ganglion cell type		Cell count
Alpha	Total [53-55]	6.7×10^{3}
	ON (45%, [56])	3.0×10^{3} [a]
	OFF (55%, [56])	3.7×10^{3} [b]
Beta [53, 56]		8.9×10^{4}
Gamma [53]		5.5×10^{3}

[a] Number derived from calculation based on percentage.
[b] Number derived from calculation based on percentage.

Supplementary Table 2

Nuclear volume, cell density, and cell counts for the visual thalamus of the cat

Anatomical structure		LGN			PGN
		A	A1	Entire A laminae	
Volume (mm^3)	Based on[57]	9.47 (47.4%)	6.88 (34.7%)	19.8	-
	Based on our measurement	9.68 (47.7%)	5.91 (29.1%)	20.3	-
Cell density (mm^{-3})	Based on[57]	2.98×10^4	2.97×10^4	-	-
	Based on our measurement	2.90×10^4	3.53×10^4	-	1.508×10^3 per unit volume of A laminae (based on[58])
Cell count	Total	2.80×10^5 Based on[57]	2.09×10^5 Based on[57]	4.89×10^5 [c] ([57])	2.35×10^4 [1.55×10^4] (number in brackets after the Abercrombie correction[59])
	X cell	-	-	2.05×10^5 [d] (42%, [60])	
	Y cell	-	-	1.71×10^5 [e] (35%, [60])	
	Interneuron	-	-	1.12×10^5 [f] (23%, [61])	

[c] Number derived from adding components.
[d] Number derived from calculation based on percentage.
[e] Number derived from calculation based on percentage.
[f] Number derived from calculation based on percentage.

SUPPLEMENTARY REFERENCES

53. Illing, R.B. & Wässle, H. The retinal projection to the thalamus in the cat: a quantitative investigation and a comparison with the retinotectal pathway. *J. Comp. Neurol.* 202, 265-285 (1981).
54. Stone, J. The number and distribution of ganglion cells in the cat's retina. *The Journal of Comparative Neurology* 180, 753-771 (1978).
55. Wässle, H., Levick, W.R. & Cleland, B.G. The distribution of the alpha type of ganglion cells in the cat's retina. *The Journal of Comparative Neurology* 159, 419-437 (1975).
56. Wässle, H., Boycott, B.B. & Illing, R.B. Morphology and mosaic of on- and off-beta cells in the cat retina and some functional considerations. *Proc. R. Soc. Lond. B Biol. Sci.* 212, 177-195 (1981).
57. Madarasz, M., Gerle, J., Hajdu, F., Somogyi, G. & Tombol, T. Quantitative histological studies on the lateral geniculate nucleus in the cat. II. Cell numbers and densities in the several layers. *J. Hirnforsch.* 19, 159-164 (1978).
58. Fitzgibbon, T. Organization of reciprocal connections between the perigeniculate nucleus and dorsal lateral geniculate nucleus in the cat: a transneuronal transport study. *Vis. Neurosci*, 19, 511-520 (2002).
59. Abercrombie, M. Estimation of nuclear population from microtome sections. *The Anatomical Record* 94, 239-247 (1946).
60. Friedlander, M.J., Lin, C.S., Stanford, L.R. & Sherman, S.M. Morphology of functionally identified neurons in lateral geniculate nucleus of the cat. *J. Neurophysiol.* 46, 80-129 (1981).
61. Montero, V.M. & Zempel, J. Evidence for two types of GABA-containing interneurons in the A-laminae of the cat lateral geniculate nucleus: a double-label HRP and GABA-immunocytochemical study. *Exp. Brain Res.* 60, 603-609 (1985).
